# Protein Kinase C Epsilon Overexpression Protects the Heart Against Doxorubicin-Induced Cardiotoxicity Via Activating SIRT1

**DOI:** 10.1007/s12012-025-09995-1

**Published:** 2025-05-06

**Authors:** Danyong Liu, Chunyan Wang, Yao Chen, Xiaolei Huang, Yajie Wen, Shan Duan, Yin Cai, Xia Li, Jianfeng He, Kaijia Han, Ting Li, Yuantao Li, Zhengyuan Xia

**Affiliations:** 1 Department of Anesthesiology, Affiliated Hospital of Guangdong Medical University, Zhanjiang, 524000 Guangdong China; 2 Department of Anesthesiology, Shenzhen Maternity and Child Healthcare Hospital, Shenzhen, 518038 Guangdong China; 3 Department of Obstetrics, Shenzhen Maternity and Child Healthcare Hospital, Shenzhen, 518038 Guangdong China; 4 Shenzhen Maternity and Child Healthcare Hospital, Shenzhen, 518038 Guangdong China; 5 Department of Health Technology and Informatics, the Hong Kong Polytechnic University, Hong Kong, China; 6 Department of Anesthesiology, Renmin Hospital of Wuhan University, Wuhan, 430060 Hubei China; 7 State Key Laboratory of Pharmaceutical Biotechnology, Department of Medicine, The University of Hong Kong, Pok Fu Lam Road, Hong Kong, China; 8 Doctoral Training Platform for Research and Translation, BoShiWan, GuanChong Village, Shuanghe Town, ZhongXiang, 431913 Hubei China

**Keywords:** Doxorubicin, Cardiotoxicity, PKC-ε, SIRT1, Apoptosis, ROS

## Abstract

Doxorubicin (DOX)-induced cardiotoxicity (DIC) is known to be associated with reduction of cardiac protein kinase C epsilon (PKC-ε). PKC-ε promotes cell survival and protects hearts against various stresses. However, it is unclear whether or not the reduction in cardiac PKC-ε expression plays a causal role in DIC and in particular the potential underlying mechanism whereby PKC-ε may protect against DIC. C57BL/6 mice (8–10-week-old) were either treated with DOX administered intraperitoneally for a duration of 4 weeks to produce cardiotoxicity, or untreated in which mice received the same volume of saline. In vitro, neonatal rat ventricle cardiomyocytes were exposed to DOX for 24 h in the absence or presence of adenovirus overexpressing PKC-ε. Cardiomyocytes in a subgroup were treated with sirtuin-1 (SIRT1) selective inhibitor Ex527. Four weeks after DOX, cardiac contractile function was decreased concomitant with increased serum CK-MB and LDH levels as well as increases in Bax-to-Bcl-2 ratio and Cleaved Caspase 3 proteins expression, while PKC-ε and Sirt1 protein expressions were significantly decreased. In vitro, DOX reduced cardiomyocyte PKC-ε and SIRT1 protein expression, decreased cardiomyocyte viability, and increased LDH release with concomitant increases in oxidative stress and apoptosis. These changes were attenuated by overexpression of PKC-ε. IP study showed that PKC-ε could directly or indirectly bind SIRT1 in cardiomyocytes, and the protect effects of PKC-ε were further canceled by SIRT1 inhibition. In conclusion, activating SIRT1 may represent a major mechanism whereby PKC-ε protects the heart against DOX-induced cell apoptosis and oxidative stress.

**Graphical Abstract:**

Dox induces cardiotoxicity via inhibiting PKC-epsilon/Sirt1 signaling which can be reversed by PKC-epsilon overexpression.

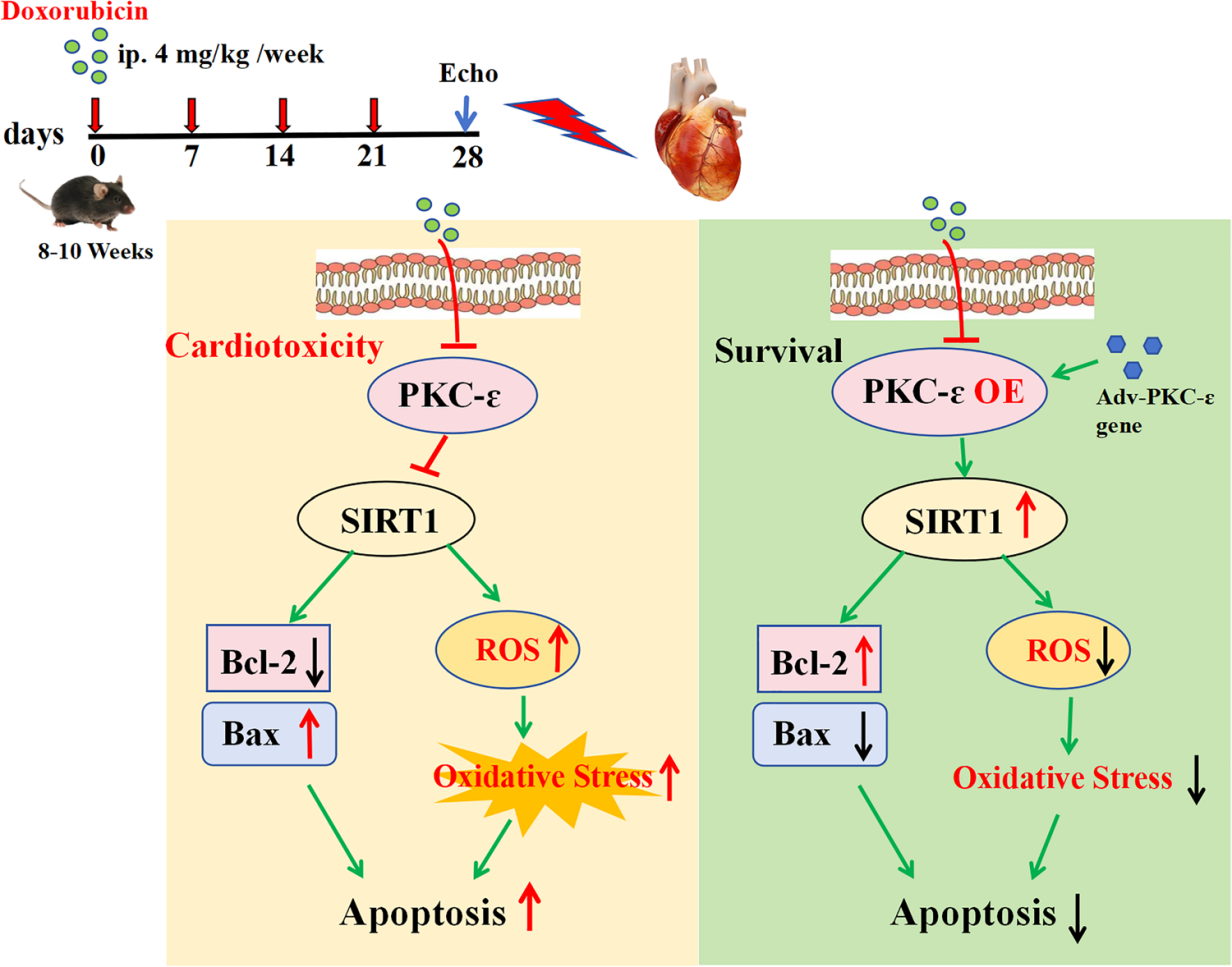

**Supplementary Information:**

The online version contains supplementary material available at 10.1007/s12012-025-09995-1.

## Introduction

Doxorubicin (DOX) is the most widely used clinical chemotherapy drug, which inhibits the proliferation of cancer cells and triggers apoptosis by inhibiting the activity of topoisomerase II and generating DNA break [2]. Unfortunately, DOX can cause cumulative and dose-dependent cardiotoxicity [20, 35, 46], characterized by the increases in vulnerability to heart failure and arrhythmias [1, 46]. Studies have shown that doxorubicin can induce cardiomyocytes to produce a large number of Reactive Oxygen Species (ROS) [44] and ferroptosis [49], thus aggravating oxidative stress and apoptosis of cardiomyocytes [38]. Therefore, there is an urgent and unmet clinical need for effective treatment to eliminate the adverse outcomes in the heart caused by DOX in cancer patients.

Pharmacological treatments that can attenuate DIC have been shown to be accompanied with an increase in the expression of protein kinase C epsilon (PKC-ε) [5, 28]. However, whether or not enhancement in cardiac PKC-ε expression may represent a mechanism by which pharmacological interventions protect against DIC is unclear. PKC-ε is a pro-survival kinase that mediates the cardioprotective effects of a variety of conditioning cardioprotective interventions. Studies have shown that PKC-ε promotes cell survival in a variety of cell types by activating the pro-survival Akt pathway [4, 16]. Thus, reduced expression of cardiac PKC-ε protein may be associated with DOX-induced cardiac injury. However, whether or not reduced expression of cardiac PKC-ε protein may play a causal role in DIC remains unclear, and in particular, the potential underlying mechanism whereby PKC-ε may protect against DIC has not been studied.

In addition, the study of John et al. showed that ischemic preconditioning of neuronal cells can achieve protective effects through PKC-ε activation of Sirtuin-1 (SIRT1) expression [34]. SIRT1 is a well-known stress response protein that plays a key role in different cellular and physiological functions [18] and has been shown to enhance cellular resistance to DIC [33, 37]. Activation of SIRT1 reduced DOX-induced oxidative stress, apoptosis, and cardiac dysfunction [14, 45]. However, it is unclear whether or not there exists a regulatory effect of PKC-ε on SIRT1 in the hearts, particularly in DIC. We hypothesized that decreased cardiac PKC-ε is responsible for DIC and that increased expression of PKC-ε can attenuate DOX-induced cardiac injury through activating SIRT1.

## Methods and Materials

### Animals and Treatments

All animal experiments were approved by the Animal Care and Use Committee of the Peking University Shenzhen Hospital and performed in compliance with the Guidelines for Care and Use of Laboratory Animals published by the US National Institutes of Health (NIH Publication No.85-23, revised 1996). Eight-to-ten-week-old C57BL/6 mice were obtained from the Gempharmatech Co., Ltd (Guangzhou, China), and kept in a specific pathogen-free barrier system with free access to the standard laboratory chow diet. The mice were allowed to be adaptive food and environment for a week before being randomly assigned to drug treatment group in which DOX (4 mg/kg once per week) was given via intraperitoneal injection for a duration of 4 weeks to produce cardiotoxicity, or to control group in which mice were injected with same volume of normal saline (NS).

### Echocardiography

Transthoracic echocardiography was performed noninvasively using a Vevo 2100 high-resolution imaging system equipped with a 30-MHz probe (MS550D; VisualSonics, Ontario, Canada). The mice were anesthetized with 1% isoflurane and maintained on a heating pad with electrocardiogram recording. M-mode echocardiograms were obtained from parasternal short-axis view at the papillary level for measurements of LV end-diastolic diameter (LVID-d) and LV mass. LV ejection fraction (EF) was calculated to evaluate cardiac systolic function. The apical four chamber view was utilized to assess the ratio of the early (E) to late (A) ventricular filling velocities (E/A ratio). All parameters were averaged over 5 cardiac cycles for analysis as previously described [6].

### Studies on Isolated Ventricular Myocytes in Primary Culture

Cardiomyocytes were isolated from the myocardium of neonatal Wistar rats (1–3 days old) and primarily cultured. After dissociation of cardiomyocytes from the heart tissue with trypsin, cells were pre-plated for 2 h(h) into culture flacons in Dulbecco’s modified Eagle medium (DMEM) with 20% newborn calf serum (NCS) to reduce the number of non-myocyte cells. Cells that did not attach to the pre-plated flacons were reseeded at a density of 1 × 10^5^ cells/mm^2^ into culture plates. 0.1 mmol/l of BRDU was also added to the medium for the first 48 h to inhibit the proliferation of non-cardiomyocytes as previously described [40]. Cultured cardiomyocytes were maintained in humidified air with 5% CO2 at 37 °C. Neonatal rat ventricle cardiomyocytes (NRVMs) were exposed to the culture medium in the presence or absence of 1 μM DOX for 24 h [19]. For SIRT1 inhibition, NRVM cells were pretreated with Ex527 (10 μM) for 6 h [25].

### Analysis of Cell Viability

The viability of cells was assessed with a Cell counting kit-8(CCK-8) kit following the instructions provided by the supplier. The treated cells were adjusted to a density of 1 × 10^5^/mL, and were then inoculated with 96-well plates and cultured at the temperature of 37℃ under 95% air and 5% CO_2_. CCK-8 assay solution was added into each well and then the cells were further cultured for 1.5 h. An enzyme marker (Thermo, USA) was used to determine the absorbance at 450 nm wavelength.

### Measurement of LDH Activity

LDH, one of the major indicators of myocardial injury, was used as cell cardiomyocyte injury index. The levels of LDH in NRVM culture mediums were measured by LDH Cytotoxicity Assay Kits followed the manufacturer’s protocols.

### TUNEL Assay

Apoptotic cell death was assessed using terminal transferase UTP nick end labeling (TUNEL). The PBS solution was used to wash the cells, and cells were then fixed with 4% paraformaldehyde for a duration of 30 min. Following PBS washing, the cell samples were added 0.3% Triton X-100 PBS mixture and incubated at room temperature for 5 min. Then, we added 50 μL of TUNEL detection solution prepared in advance to the samples, and they were incubated for 60 min at 37 °C in the dark. Further, we added DAPI to mount the slide and observed under a fluorescence microscope.

### DHE Staining

Reactive oxygen species production was assessed with Dihydroethidium(DHE) staining following manufacturer’s instruction. Red fluorescence was emitted when DHE was oxidized by superoxide, and it was detected by fluorescence microscopy (Olympus, Japan) as previously described [12].

### Quantification of MDA

Samples of the hearts or cultured cells were collected and homogenized in pPBS. And, Malondialdehyde (MDA) assay kit was used to assess the amount of MDA production.

### PKC-ε Gene Overexpression Studies in NRVMs

In in vitro* experiments*, we aimed to confirm the protective effect of PKC-ε gene overexpression. PKC-ε gene (OBiO Technology, Shanghai Corp.,Ltd) knock up was applied to increase PKC-ε expression following the manufacturer’s protocols. We seeded and assigned 2 × 10^5^ cells each into 5 treatment groups composed as follows: (1) Normal control (NC); (2) NC + Doxorubicin (DOX):1uM DOX for 24 h; (3) NC + Adv-PKC-ε; (4) NC + Adv-PKC-ε + DOX; (5) NC + Adv-PKC-ε + Ex527 + DOX. With respect to control adenovirus or adenovirus-overexpressed PKC-ε gene treatment groups, cells were exposed to 1 umol/L DOX for 24 h and then we collected cells and mediums for further analyses.

### Wheat Germ Lectin (WGA) Staining

Animal hearts were paraffin-embedded after fixation in 4% buffered formaldehyde for 24 h and sectioned into slices at 5-µm thick each. The cross-sectional areas of the cardiomyocytes in the left ventricle (LV) were observed by fluorescein-conjugated wheat germ agglutinin (WGA;5 µg/mL,AAT Bioquest,USA) staining and evaluated by calculating the single myocyte cross-sectional areas measured by ImageJ software (National Institutes of Health, USA).

### F-Actin Staining

The Phalloidin-iFluor 488 staining kit was employed for staining actin filaments (F-actin). Cardiomyocytes in culture were fixed with 4% paraformaldehyde for 10 min, and were permeabilized with 0.2% Triton X-100 for 30 min, before being blocked with 2.5% bovine serum albumin for a duration of 30 min. The nucleus and actin filaments were visualized with DAPI (Beyotime, shanghai, China) and iFluor 488-conjugated phalloidin, respectively. A confocal laser scanning microscope (Olympus, Japan) was used to obtain fluorescence images. The cell area was assessed with ImageJ software (National Institutes of Health, USA).

### Immunoprecipitation

To immunoprecipitate endogenous proteins extracted from cells homogenates, 100 µg of protein was incubated with commercial primary antibodies against PKC-ɛ or control IgG for 16 h at 4 °C in a rotating incubator. The immune complexes were collected by incubation with True-Blot IP beads (eBioscience, Hatfield, United Kingdom) for another 2 h. The cultured cell samples were rinsed with lysis buffer for three times and then eluted. The SIRT1 protein level was assessed by Western blot with anti-SIRT1 antibodies, as described [17].

### Western Blotting

Heart tissues and cell lysates were prepared using a lysis buffer (Cell Signaling Technology) supplemented with Protease Inhibitor Cocktail (Roche, Mannheim, Germany) and Phosphatase Inhibitor Cocktail (Roche). Equal denatured protein lysates from cells or heart tissues were separated by 8%−12% sodium dodecyl sulfate polyacrylamide gel electrophoresis and transferred onto polyvinylidene difluoride membranes. The primary antibodies against PKC-ɛ (1:1000), SIRT1 (1:1000), Bcl-2 (1:1000), Bax (1:1000), Cleaved Caspase 3 (1:500), and GAPDH (1:1000) were purchased from Cell Signaling Technology, as was horseradish peroxidase-conjugated anti-mouse (1:5000) or anti-rabbit (1:5000) secondary antibodies. Enhanced chemiluminescent substrate (WBKLS0500, Millipore) was used to visualize the protein bands and ImageJ software 18.0 (National Institutes of Health, Bethesda,USA) was employed to quantify levels of protein expression.

### Statistics

Results of the current study are presented as mean ± standard error of the mean (S.E.M). The between-groups comparisons were carried out either by unpaired Student’s t test, or one-way ANOVA followed by Bonferroni test, where appropriate (GraphPad Prism 8.0, San Diego, USA). P value < 0.05 was considered as statistically significant.

## Results

### DOX Induces Cardiac Injury and Dysfunction in Mice

**Fig. 1:**
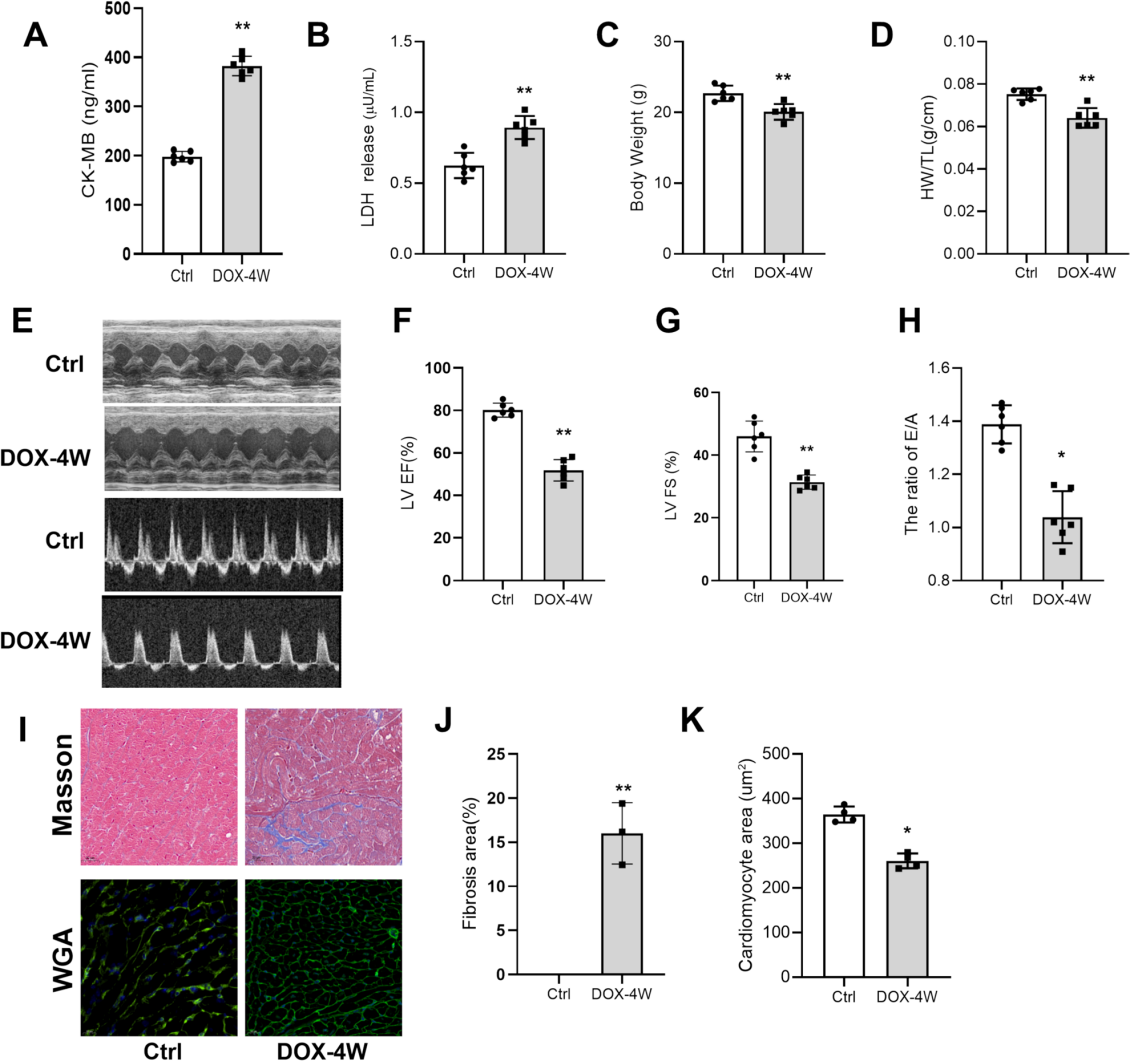
DOX-induced cardiac injury and dysfunction in mice. **A** Serum CK-MB levels. **B** Serum LDH levels. **C** Mice body weight. **D** Ratio of heart weight to tibial length in mice. **E**–**H** Ultrasonic examination of mouse heart: EF% and FS value of systolic function and E/A ratio of diastolic function. **I** Masson staining and WGA staining; scale bar: 20 μm. **J** Quantitative analysis of cardiac fibrosis area by Masson staining. **K** Quantitative analysis of myocyte cross-sectional area by WGA staining. HW/TL: Ratio of heart weight to tibial length. All data are expressed as mean ± S.E.M (n = 4–6). *P < 0.05 vs Ctrl group, **P < 0.01

Mouse DIC model was established by injecting DOX (4 mg/kg once per week, i.p.) for 4 weeks. Compared to control mice (injected with the same volume of saline), myocardial injury was obvious in the DOX treatment group, manifested as significant increases in CK-MB and LDH (Fig. 1A, B); meanwhile, the body weight and the ratio of heart weight to tibia length of mice with DIC were also found to be significantly decreased (Fig. 1C, D). The results of cardiac function tests showed that the systolic and diastolic functions of the mice were significantly reduced (Fig. 1E–H). Moreover, Cell nuclei appeared as blue dominant dots due to DIC, while normal cell nuclei were navy-blue (Fig. 1 I), and the results of WGA staining showed that the cross-sectional area of cardiomyocytes was reduced in the DOX-treated group(Fig. 1J, K). These observations suggested that DOX treatment induces cardiac injury and dysfunction in mice.

### DOX Decreased Myocardial Expression Levels of PKC-ɛ and SIRT1 Proteins, Accompanied by Induced Oxidative Stress and Apoptosis in Mice

**Fig. 2:**
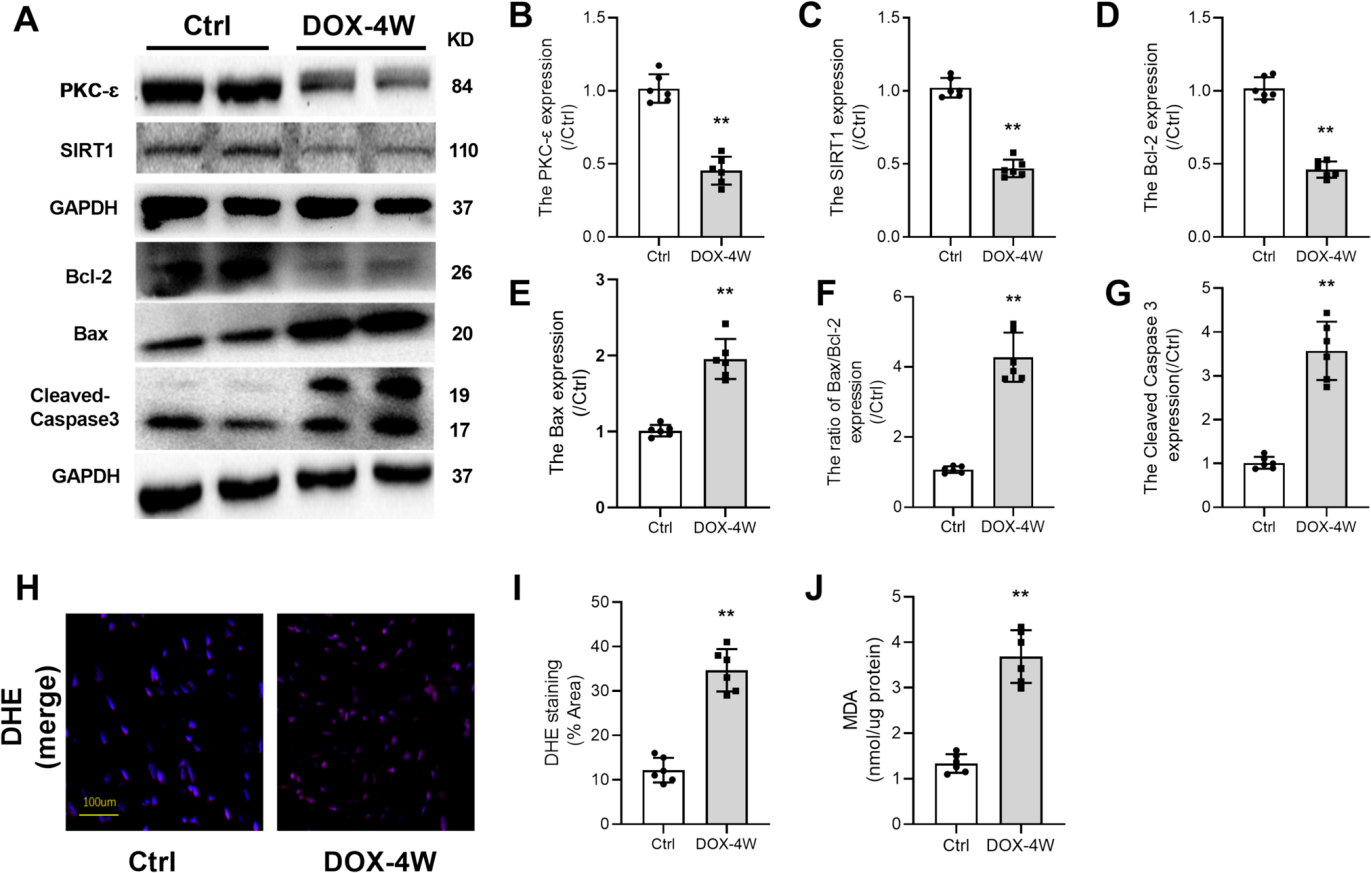
DOX-induced heart oxidative stress and apoptosis in mice. **A** The expressions of PKC-ɛ, SIRT1, Bcl-2, Bax, and Cleaved Caspase3 in myocardial tissue were detected by Western blotting. **B** The expression level of PKC-ɛ protein. **C** The expression level of SIRT1 protein. **D** The expression level of Bcl-2 protein. **E** The expression level of Bax protein. **F** The ratio of Bax to Bcl-2. **G** The expression level of Cleaved Caspase3 protein. **H**, **I** Analysis of ROS production by DHE staining; scale bar: 100 μm. **J** Determination of MDA content. All data are expressed as mean ± S.E.M (n = 4–6). *P < 0.05 vs Ctrl group, **P < 0.01

As shown in Fig. 2, compared with the control group, the expressions of PKC-ɛ, SIRT1, and Bcl-2 in the DOX-treated group were significantly decreased (Fig. 2A–D), while expressions of apoptosis-related proteins (Bax, Bax/Bcl-2,Cleaved Caspase3) were significantly increased (Fig. 2E–G). Meanwhile, ROS production and MDA content also increased significantly (Fig. 2H–J) (all P < 0.05 vs. Ctrl). These results indicated that PKC-ɛ and SIRT1 may be associated with DIC.

### Doxorubicin-Induced Reduction in the Protein Expressions of PKC-ɛ and SIRT1 in NRVMs was Accompanied by Induced Oxidative Stress and Apoptosis

**Fig. 3:**
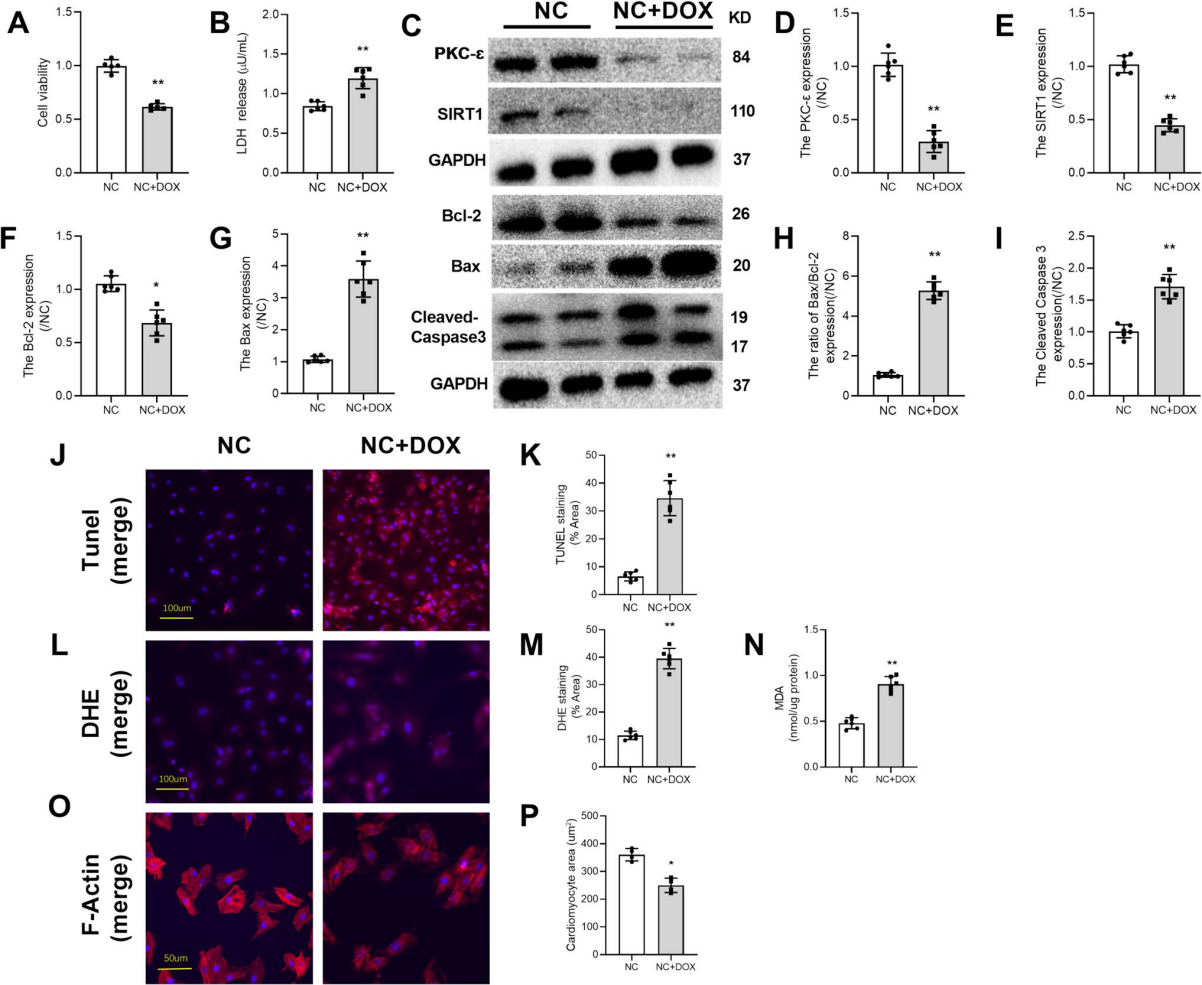
DOX decreased the expressions of PKC-ɛ and SIRT1 in NRVMs, promoted oxidative stress and apoptosis. **A** Detection of Cell viability. **B** Detection of LDH. **C** The expressions of PKC-ɛ, SIRT1, Bcl-2, Bax, and Cleaved Caspase3 in NRVMs were detected by Western blotting. **D** The expression level of PKC-ɛ protein. **E** The expression level of SIRT1 protein. **F** The expression level of Bcl-2 protein. **G** The expression level of Bax protein. **H** The ratio of Bax to Bcl-2. **I** The expression level of Cleaved Caspase3 protein. **J**, **K** Analysis of apoptosis by TUNEL staining; scale bar: 100 μm. **L**, **M** Analysis of ROS production by DHE staining; scale bar: 100 μm. **N** Determination of MDA content. **O**, **P** The cell skeleton and nucleus were stained using the Phalloidin-iFluor 488 reagent or DAPI. Red fluorescence indicates actin filaments, whereas blue fluorescence indicates the nuclei; scale bar: 50 μm. All data are expressed as mean ± S.E.M (n = 3–6). *P < 0.05 vs NC group, **P < 0.01

In in vitro* experiments*, cells were exposed to DOX (1 μM) for 24 h. Compared with the control (NC) group, DOX treatment decreased cell viability and increased LDH release(Fig. 3A, B), and the expressions of PKC-ɛ, SIRT1, and Bcl-2 were significantly decreased (Fig. 3C–F), while expressions of apoptosis-related proteins (Bax, Bax/Bcl-2, Cleaved Caspase3) were significantly increased that was accompanied with increased TUNEL-positive cells(Fig. 3G–J). In addition, DOX exposure increased ROS production and oxidative stress manifested as increased DHE intensity and increased MDA content (Fig. 3L–N). This increase in oxidative stress was associated with reduced cell size measured by F-actin Phalloidin staining (Fig. 3O, P). These results illustrated that PKC-ɛ and SIRT1 may play roles in DOX-induced cell apoptosis and oxidative stress.

### PKC-ɛ May be a Key Upstream Factor of SIRT1

**Fig. 4:**
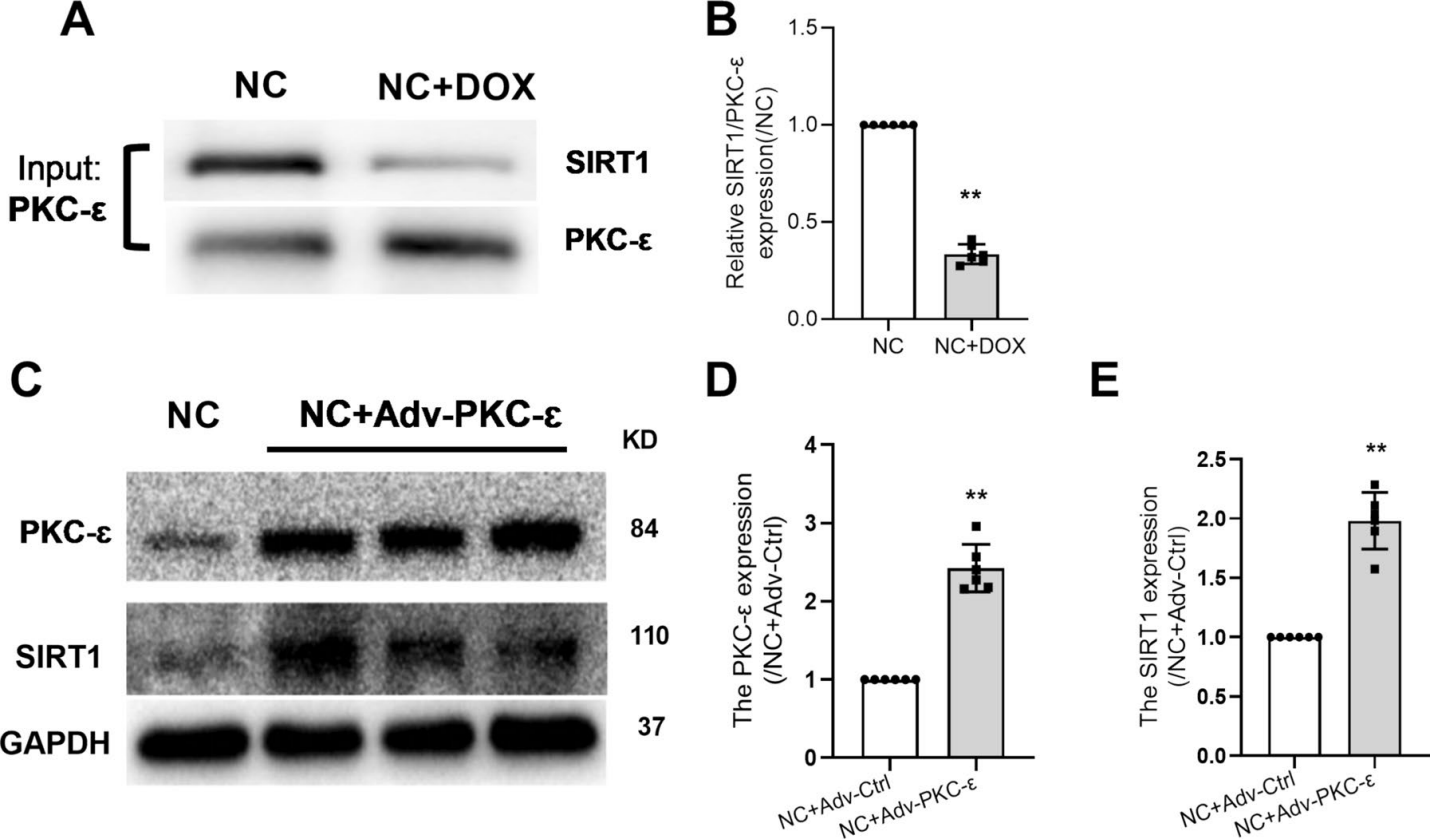
PKC-ɛ may be a key upstream factor of SIRT1. **A**, **B** Co-IP PKC-ε and SIRT1 in primary cardiomyocytes. The relative density of NC + DOX group was normalized against the control group (NC). **C** Western blotting detected the expression of PKC-ε and SIRT1 in NRVMs. **D** Expression level of PKC-ε protein in NRVM cells after transfecting adenovirus-overexpressed PKC-ε gene. **E** Expression level of SIRT1 protein in NRVM cells after transfecting adenovirus-overexpressed PKC-ε gene. The relative density of NC + Adv-PKC-ε group was normalized against the NC + Adv-Ctrl group (NC + Adv-Ctrl). All data are expressed as mean ± S.E.M (n = 6). *P < 0.05 vs NC + Adv-Ctrl group, **P < 0.01

To examine the possible interaction between PCK-ε and SIRT1, IP was performed by pulldown PKC-ε in protein lysate from NRVMs. As shown in Fig. 4, SIRT1 protein was detectable in complex pulldown by PKC-ε antibody (Fig. 4A), suggesting direct or indirect binding of PKC-ε and SIRT1 in cardiomyocytes. Also, protein expression of SIRT1 detected in PKC-ε pulldown was reduced after DOX treatment (Fig. 4B). Interestingly, adenovirus overexpression of PKC-ε increased SIRT1 protein expression in NRVMs (Fig. 4C–E). These results showed that PKC-ε regulates SIRT1 in cardiomyocytes by direct or indirect binding.

### Overexpression of PKC-ε Attenuated DOX-Induced Apoptosis and Oxidative Stress

**Fig. 5:**
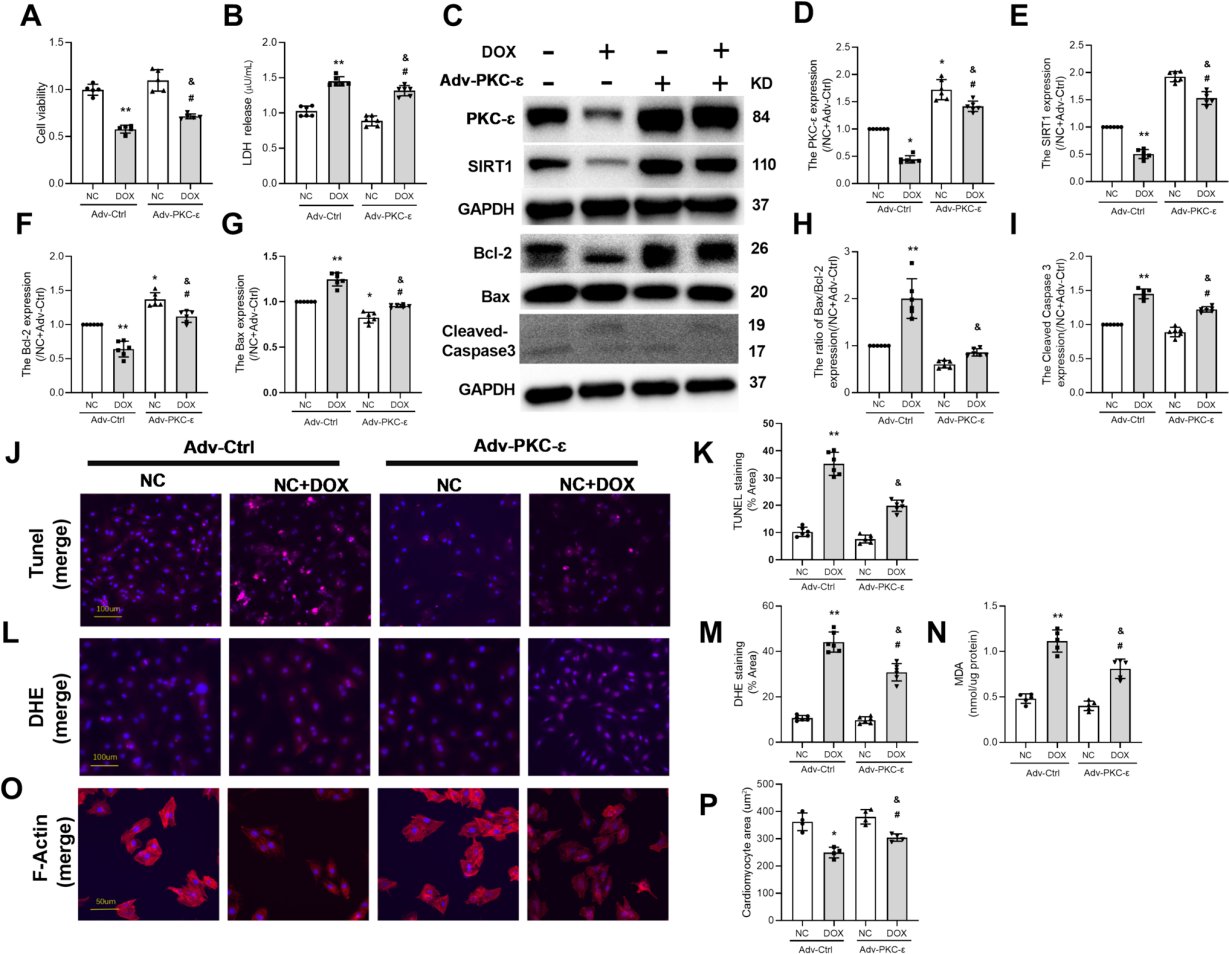
Overexpression of PKC-ε decreased the cardiomyocyte injury induced by DOX. **A** Detection of Cell viability. **B** Detection of LDH. **C** The expressions of PKC-ɛ, SIRT1, Bcl-2, Bax, and Cleaved Caspase3 were detected by Western blotting. **D** The expression level of PKC-ɛ protein. **E** The expression level of SIRT1 protein. **F** The expression level of Bcl-2 protein. **G** The expression level of Bax protein. **H** The ratio of Bax to Bcl-2. **I** The expression level of Cleaved Caspase3 protein. **J**, **K** Analysis of apoptosis by TUNEL staining; scale bar: 100 μm. **L**, **M** Analysis of ROS production by DHE staining; scale bar: 100 μm. **N** Determination of MDA content. **O**, **P** The cell skeleton and nucleus were stained using the Phalloidin-iFluor 488 reagent or DAPI. Red fluorescence indicates actin filaments, whereas blue fluorescence indicates the nuclei; scale bar: 50 μm. The relative densities of all other groups were normalized against the NC + Adv-Ctrl group (NC + Adv-Ctrl). All data are expressed as mean ± S.E.M (n = 5–6). *P < 0.05 vs. NC + Adv-Ctrl group, ^&^*P* < 0.05 vs. Adv-Ctrl + DOX group, ^#^*P* < 0.05 vs. NC + Adv-PKC-ε group, **P < 0.01 vs. NC + Adv-Ctrl group

To examine the role of PKC-ε in DOX-induced cardiomyocyte apoptosis, NRVMs were treated with DOX without or with adenovirus overexpression of PKC-ε. Consistent with our previous findings, DOX treatment decreased expressions of PKC-ε, SIRT1, and Bcl-2 protein(Fig. 5C–F), while decreased cell viability(Fig. 5A) and increased LDH release(Fig. 5B) as well as enhanced Bax-to-Bcl-2 ratio and Cleaved Caspase3 protein expression (Fig. 5G–I), leading to increased TUNEL-positive cells (Fig. 5J, K). DOX treatment also increased ROS production and oxidative stress (Fig. 5L–N) as well as decreased cell size in NRVMs (Fig. 5O, P). Compared with the Adv-Ctrl + DOX group, adenovirus therapy in the Adv-PKC-ε + DOX group resulted in significantly increased expressions of PKC-ε and SIRT1 protein (Fig. 5C–D), while reducing DOX-induced apoptosis and oxidative stress (Fig. 5J–N), and restoring the viability (Fig. 5A) and size of primary cardiomyocytes (Fig. 5O, P)(all *P* < 0.05 vs. Adv-Ctrl + DOX). These data indicated that PKC-ε is sufficient to protect cardiomyocytes against DIC.

### SIRT1 Inhibition Canceled the Protective Effects of PKC-ɛ Overexpression Against DIC

**Fig. 6:**
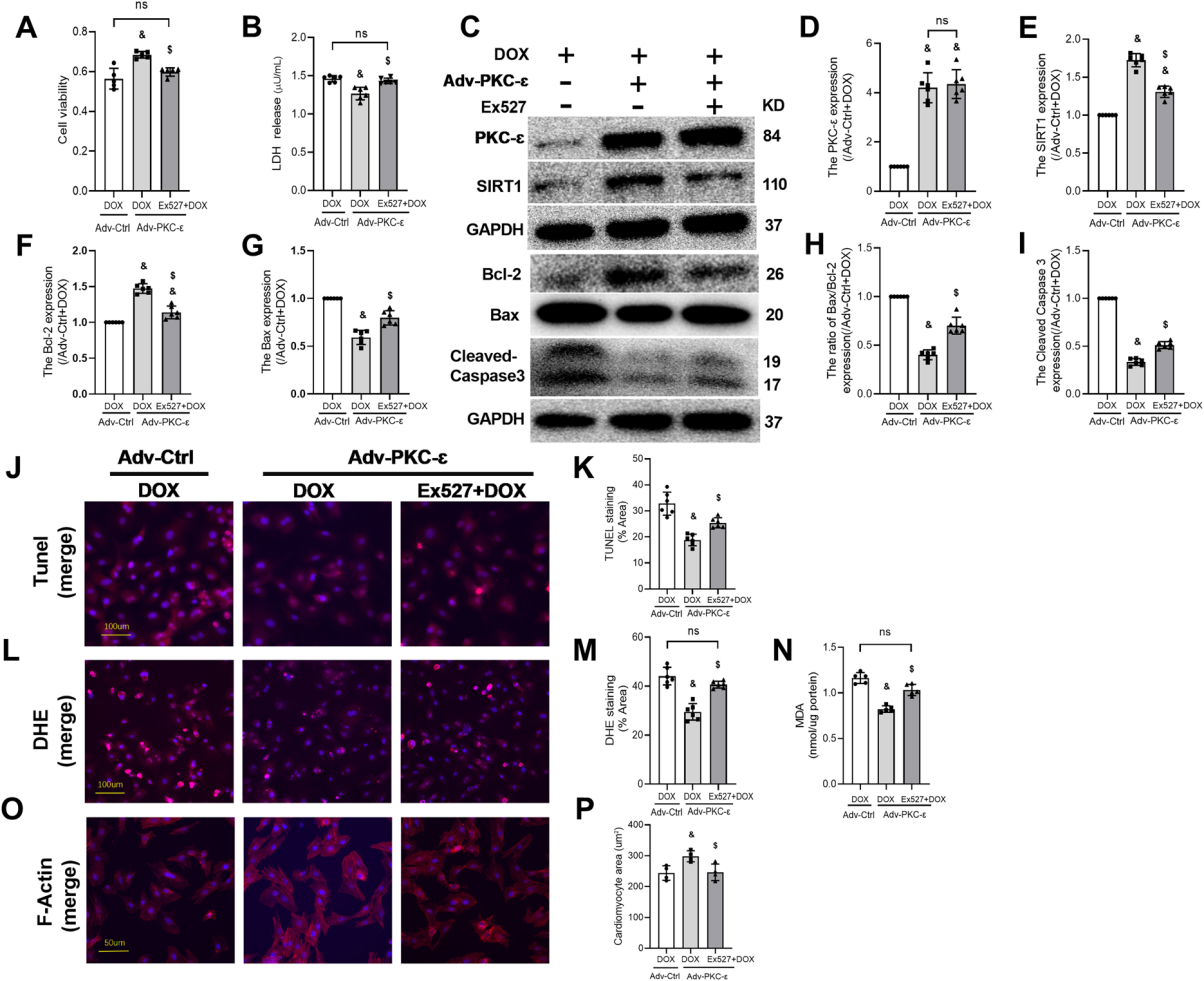
PKC-ε attenuated DOX-induced oxidative stress and apoptosis by activating SIRT1. **A** Detection of Cell viability. **B** Detection of LDH. **C** The expressions of PKC-ɛ, SIRT1, Bcl-2, Bax, and Cleaved Caspase3 were detected by Western blotting. **D** The expression level of PKC-ɛ protein. **E** The expression level of SIRT1 protein. **F** The expression level of Bcl-2 protein. **G** The expression level of Bax protein. **H** The ratio of Bax to Bcl-2. **I** The expression level of Cleaved Caspase3 protein. **J**, **K** Analysis of apoptosis by TUNEL staining; scale bar: 100 μm. **L**, **M** Analysis of ROS production by DHE staining; scale bar: 100 μm. **N** Determination of MDA content. **O**, **P** The cell skeleton and nucleus were stained using the Phalloidin-iFluor 488 reagent or DAPI. Red fluorescence indicates actin filaments, whereas blue fluorescence indicates the nuclei; scale bar: 50 μm. The relative densities of all other groups were normalized against the Adv-Ctrl + DOX group (Adv-Ctrl + DOX). All data are expressed as mean ± S.E.M (n = 5–6). ^&^*P* < 0.05 vs. Adv-Ctrl + DOX group; ^$^*P* < 0.05 vs. Adv-PKC-ε + EX527 + DOX group; *ns* not significant

To further examine the role of SIRT1 in PKC-ε-mediated protective effects in DOX-induced cell apoptosis, NRVMs were treated with Ex527 (specific SIRT1 inhibitor). As shown in Fig. 6, in NRVMs treated with DOX, adenovirus overexpression of PKC-ε increased SIRT1 protein expression, while inhibition of SIRT1 by Ex527 did not affect PKC-ε expression (Fig. 6C–E). In NRVMs treated with DOX, adenovirus overexpression of PKC-ε increased cell viability(Fig. 6A) and reduced LDH release (Fig. 6B) as well as reduced Bax-to-Bcl-2 ratio (Fig. 6F–H) and Cleaved Caspase 3 protein expression (Fig. 6I) and TUNEL-positive cells (Fig. 6J, K), while decreased DOX-induced ROS generation measured by DHE staining (Fig. 6L, M) and MDA production(Fig. 6N). It is suggestive that PKC-ε conferred protective effects against DOX-induced cell apoptosis. However, these protective effects of PKC-ε were canceled by SIRT1 inhibition with Ex527. These results showed that PKC-ε protects against DOX-induced cell apoptosis through SIRT1.

## Discussion

In the present study, we found that decrease in cardiac PKC-ɛ protein expression after DOX treatment was associated with increases in myocardial cell apoptosis and oxidative stress. We provide evidence that in cardiomyocytes, overexpression of PKC-ε is sufficient to protect cardiomyocytes against DOX-induced cell apoptosis and oxidative stress. We further demonstrated that PKC-ε conferred these protective effects through binding and activating SIRT1. Thus, effective means that activate PKC-ε/SIRT1 signaling pathway may serve as a potential therapeutic avenue against DIC.

Previous studies have shown that DIC involves multiple mechanisms, including increase in ROS and lipid peroxidation, calcium overload, and deterioration of mitochondrial function, DNA damage, and cardiomyocyte apoptosis [15, 30]. Consistent with this, our current study showed that mice treated with DOX exhibited increased oxidative stress and cell apoptosis. Similarly, our in vitro study conducted in NRVMs showed that DOX led to increased ROS generation and MDA production as well as apoptosis. Moreover, in our study, the cross-sectional area of cardiomyocytes decreased after DIC, a trend similar to the study of Ge et al. [21, 41]. In contrast to the study of Wang et al. [36], this difference may be due to the different species of mice used in Wang’s study. Our study finding that overexpression of PKC-ε was sufficient to attenuate these DOX-induced cell area reduction, oxidative stress, and apoptosis indicates that PKC-ε plays a key role in the mechanism of DIC.

PKC-ε belongs to the PKC family. The PKC family consists of at least ten members that have been categorized into three groups based on their structure and biochemical properties [26]. Ca^2+^ and diacylglycerol (DAG) are required for the activation of conventional or cPKCs (α, βI, βII and γ). While novel or nPKCs (δ, ε, η and θ) are dependent on DAG but not Ca^2+^, and the atypical or aPKCs (ζ and λ/ι) are independent of both Ca^2+^ and DAG [4, 26]. PKC isoenzymes are effector of DAG and major targets of phosphonate tumor promoters, playing an important role in cell cycle regulation, cell survival, malignant transformation, and cell apoptosis [9]. In many cases, altered PKC expression may be associated with disease progression [9], where isozyme alpha, epsilon, and zeta play a major role in antitumor therapy and apoptosis [31]. Studies have shown that overexpression of PKC-ε protects cells from apoptosis caused by cytokine consumption by inducing anti-apoptotic protein Bcl-2 [10], and that PKC stabilizes the expression of Bcl-2 to alleviate DOX-mediated apoptosis [8]. In line with these findings, in the current study, PKC-ε overexpression increased Bcl-2 protein expression in DOX treatment. We further provide evidence that PKC-ε does so by activating SIRT1. Although the regulation of SIRT1 by PKC-ε has been mentioned in ischemic preconditioning of the central nervous system [34], we have shown it in the heart for the first time.

SIRT1 is a member of the seven known proteins of the sirtuin(SIRT) family [7, 22]. SIRT proteins have been shown to be involved in a wide range of physiological and pathological processes, including aging, apoptosis, stress and inflammatory responses, energy efficiency, circadian clocks, and mitochondrial biogenesis [29, 43]. SIRT1 is an epigenetic enzyme that induces epigenetic changes in its downstream targets, thereby affecting biological functions [3]. It is also a key regulator of tissue homeostasis, inducing downstream protein deacetylation and promoting cell survival [39]. Previous studies have shown that activation of SIRT1 by various drugs can reduce DOX-induced oxidative stress, apoptosis, and cardiac dysfunction [14, 45]. This phenomenon was also observed in our study, we showed that DOX treatment decreased SIRT1 protein expression in cardiomyocytes, which was associated with increased ROS production, and led to excessive cell injury, oxidative stress, and apoptosis. SIRT1 is closely related to oxidative stress, and SIRT1/FOXO3a/MnSOD signal is the most important antioxidant axis, responsible for inhibiting mitochondrial oxidative stress [24]. Moreover, increased expression of SIRT1 can activate AMPK, restore protein abundance and myocardial nucleus accumulation of Nrf2, and restore the levels of downstream factors catalase (CAT), superoxide dismutase (SOD), and heme oxygenase 1(HO-1) [42]. Nrf2 is a key antioxidant sensor for maintaining redox homeostasis, and activation of SIRT1/Nrf2 signaling pathway has been shown to reduce oxidative stress, thereby inhibiting ferroptosis and reducing DIC [18]. Our study indicated that DIC is mainly caused by inhibiting the expression of PKC-ε and SIRT1 proteins, which leads to severe oxidative stress damage in cardiomyocytes. These injuries were attenuated by PKC-ε overexpression, but the protective effect of PKC-ε was further canceled by SIRT1 inhibition. These findings not only provide additional mechanistic insight of SIRT1 in mediating antioxidant capacity and cell survival in DIC, but also demonstrate PKC-ε as a new regulator of SIRT1 in the heart.

In addition, recent studies have demonstrated that other members of the SIRT family, including SIRT2 [51], SIRT3 [32], SIRT4 [13], and SIRT6 [11], also play important roles in combating against DIC. SIRT proteins can be categorized based on their subcellular localization into cytoplasmic proteins (SIRT1, 2), mitochondrial proteins (SIRT3,4,5), and nuclear proteins (SIRT1,2,6,7) [7, 22]. Due to their distinct subcellular positioning, these SIRT proteins exhibit varying mechanisms of resistance to DIC. Specifically, SIRT2 has been shown to inhibit DOX-induced apoptosis [50] and oxidative stress [51]. SIRT3 protects against DIC by maintaining mitochondrial function, reducing oxidative stress [48], and endoplasmic reticulum stress [47]. SIRT4 can inhibit DOX-induced apoptosis and autophagy [13]. SIRT6 safeguards against DIC by preserving mitochondrial function and enhancing mitochondrial autophagy [27]. Interestingly, apart from SIRT1, study on cerebral ischemic preconditioning has revealed that PKC-ε plays a protective role by regulating SIRT5 [23]. Our study has confirmed that PKC-ε can also regulate SIRT1 in the heart. This raises the question: does PKC-ε regulate other members of the SIRT family in the heart as well? If so, overexpression of PKC-ε might play a multifaceted role in reducing DIC, akin to “killing several birds with one stone.”

## Limitations of the Study

While we demonstrated that the role of PKC-ε-mediated SIRT1 activation in combating DIC, there are some limitations to this study. First, exactly how PKC-ε regulates SIRT1 activity remains unclear. Second, although we have demonstrated in vitro that activation of SIRT1 may represent a major mechanism whereby PKC-ε conferred protective effects against DIC, this has not been reconfirmed in vivo. Third, whether PKC-ε also regulates other members of the SIRT family in the heart is unclear, and it is worth further exploration. Future studies will focus on the detailed mechanisms by which PKC-ε mediates SIRT1 activation in vitro and in vivo and also investigate the downstream pathway of SIRT1 signaling in order to address role and mechanisms of PKC-ε-mediated regulation of SIRT proteins in the prevention of DIC.

## Conclusion

Our current study demonstrated that DIC may be caused by down-regulating the expression of the PKC-ε protein and thereby inhibiting the PKC-ε/SIRT1 signaling pathway, and that overexpression of PKC-ε is sufficient to protect the heart against DIC by activating SIRT1.

## Supplementary Information

Below is the link to the electronic supplementary material.

Supplementary file1 (PDF 1361 KB)

Supplementary file2 (PDF 1510 KB)

## Data Availability

Data are provided within the manuscript or supplementary information files. And the link of the original data is https://data.mendeley.com/preview/r42kd5rwzv?a = 820c987f-4756-4bee-b162-5f3894c4b274.
