# Supplementary figures and images for: Protein Kinase C Epsilon Overexpression Protects the Heart Against Doxorubicin-Induced Cardiotoxicity Via Activating SIRT1
Source: Cardiovasc Toxicol. 2025 May 6;25(6):915–28. doi: 10.1007/s12012-025-09995-1 (PMC12116906; doi:10.1007/s12012-025-09995-1)

Fig2

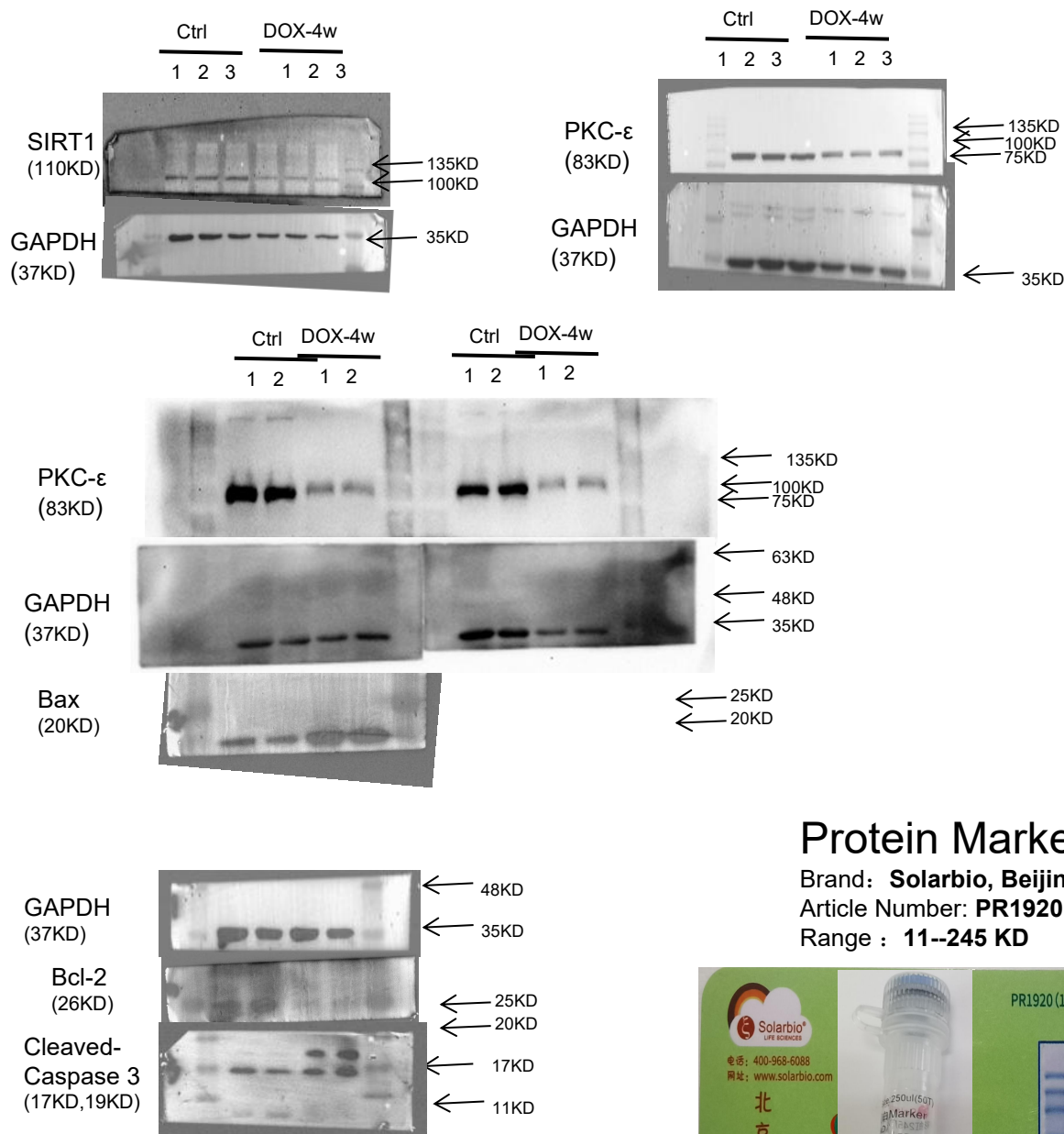

## Protein Marker

Brand: **Solarbio, Beijing**  
Article Number: **PR1920**  
Range : **11--245 KD**

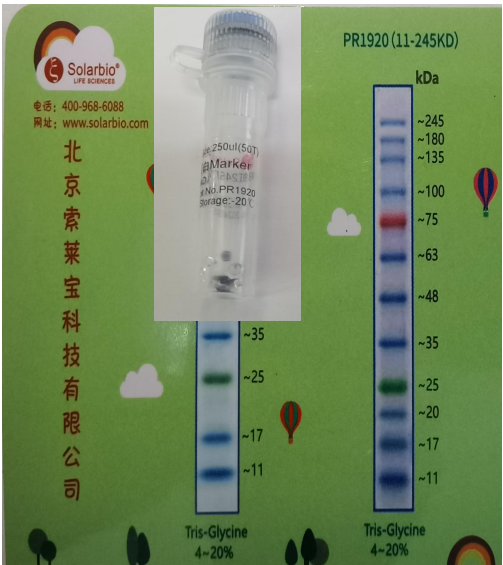

Fig3

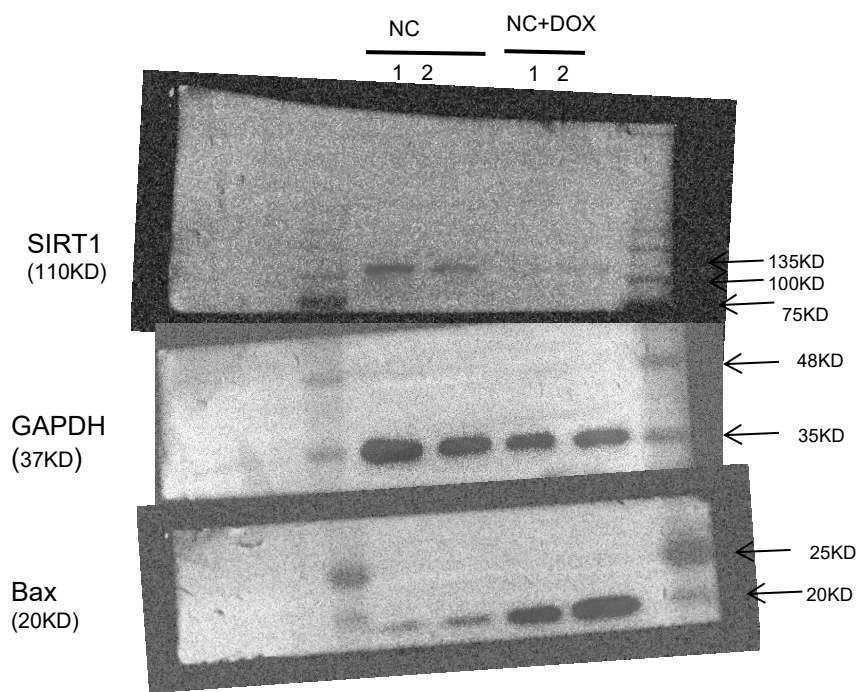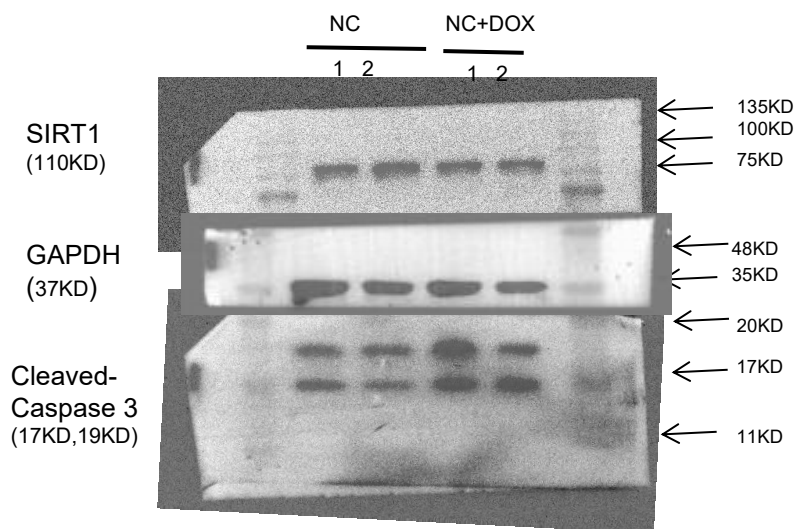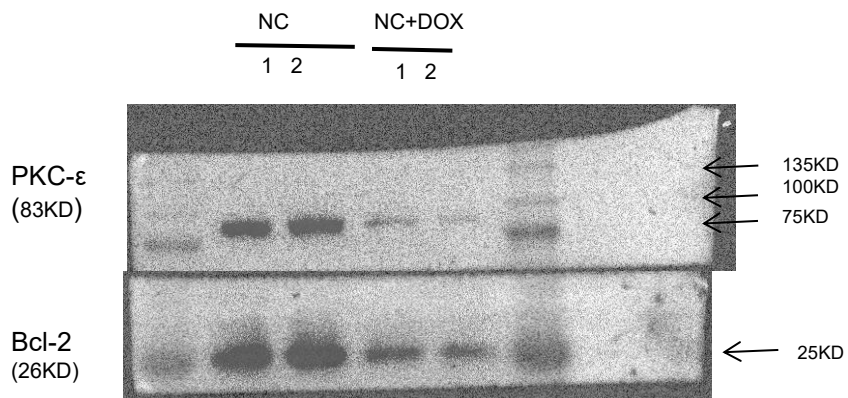

Fig4

IP

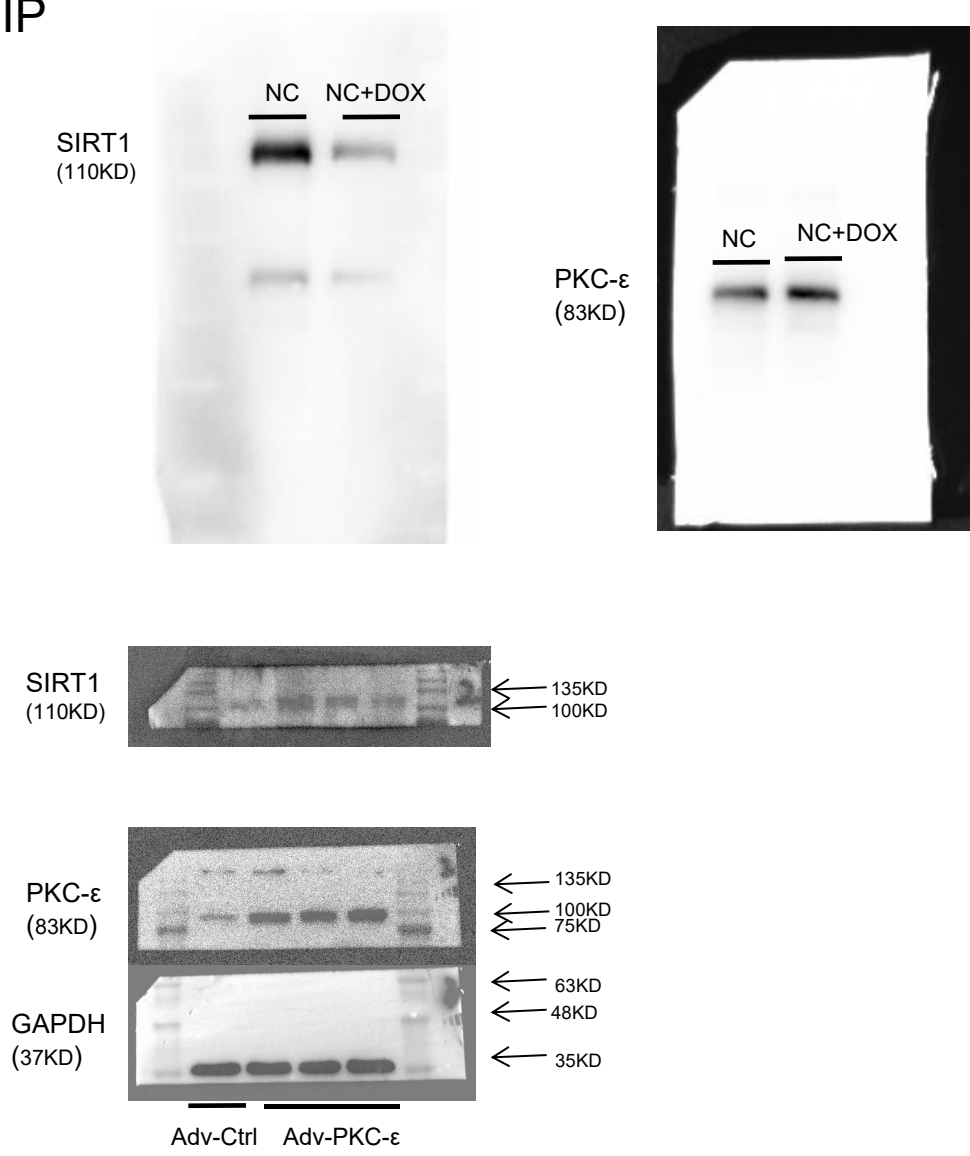

Fig5

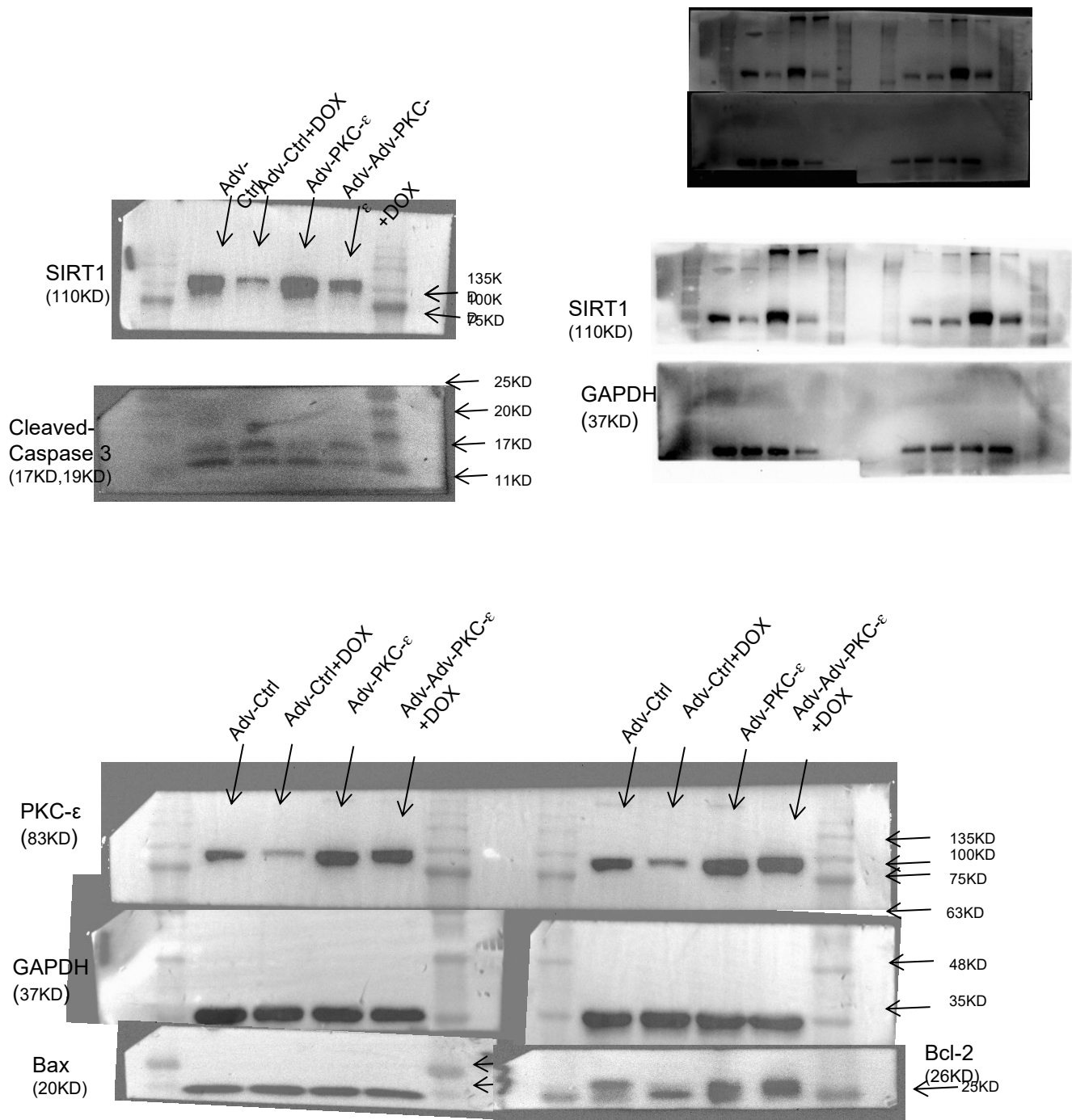

**Fig6**

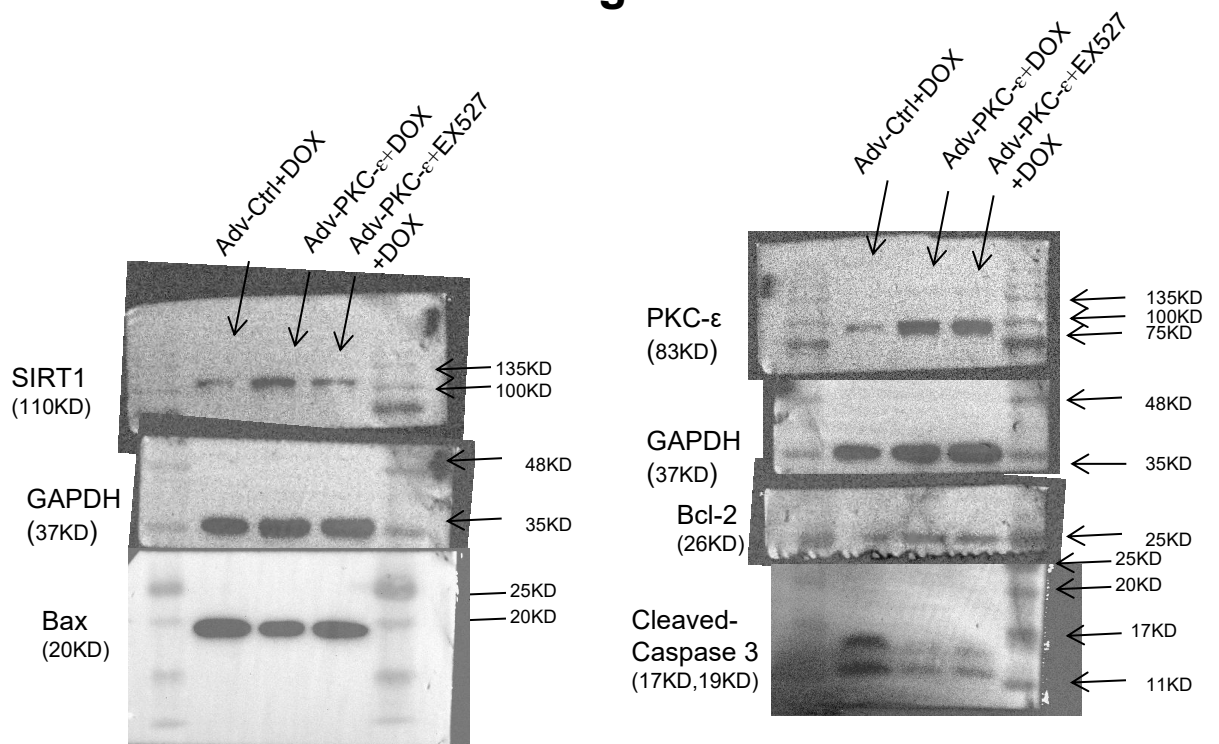

Supplement: Supplementary file 1 — Supplementary file1 (PDF 1361 KB) [file 12012_2025_9995_MOESM1_ESM.pdf]

**Fig2**

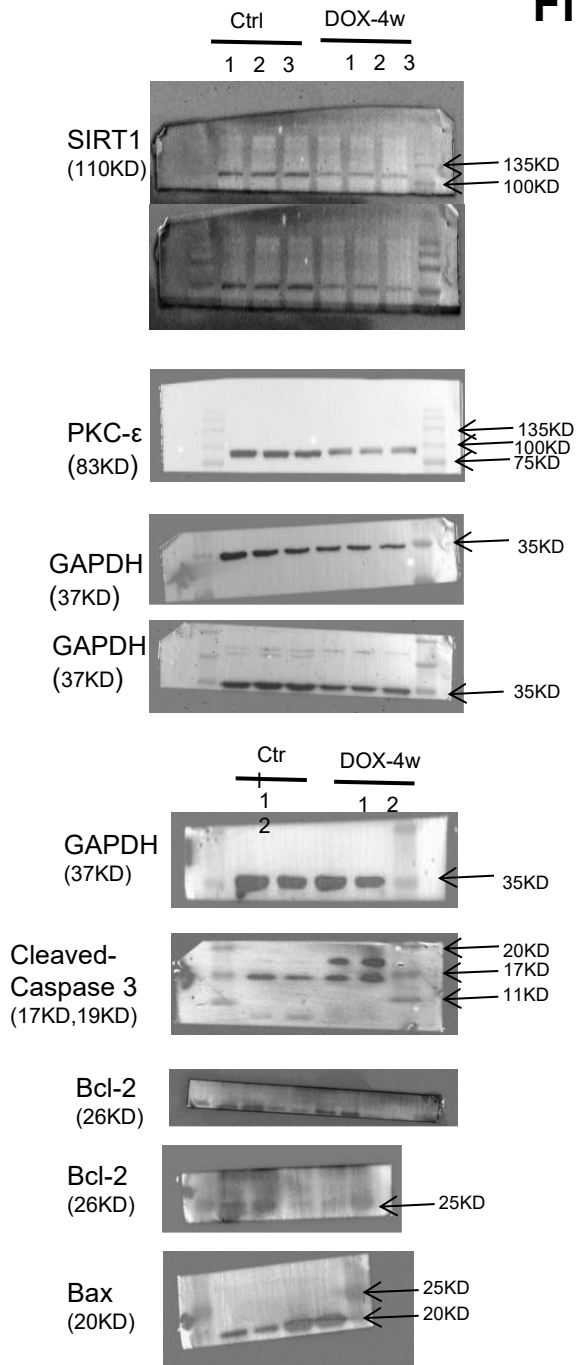

## Protein Marker

Brand: **Solarbio, Beijing**  
Article Number: **PR1920**  
Range : **11--245 KD**

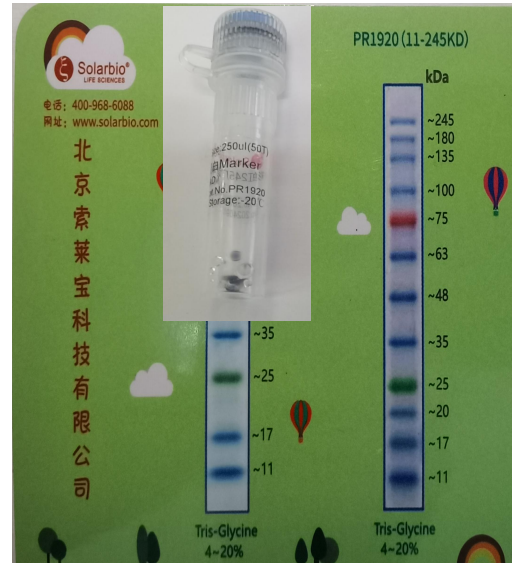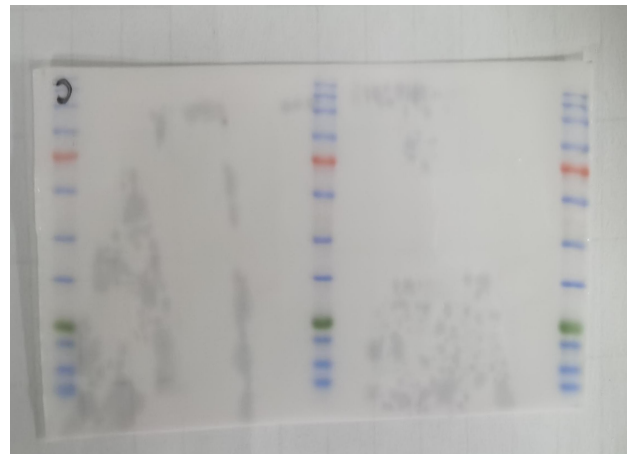

Fig3

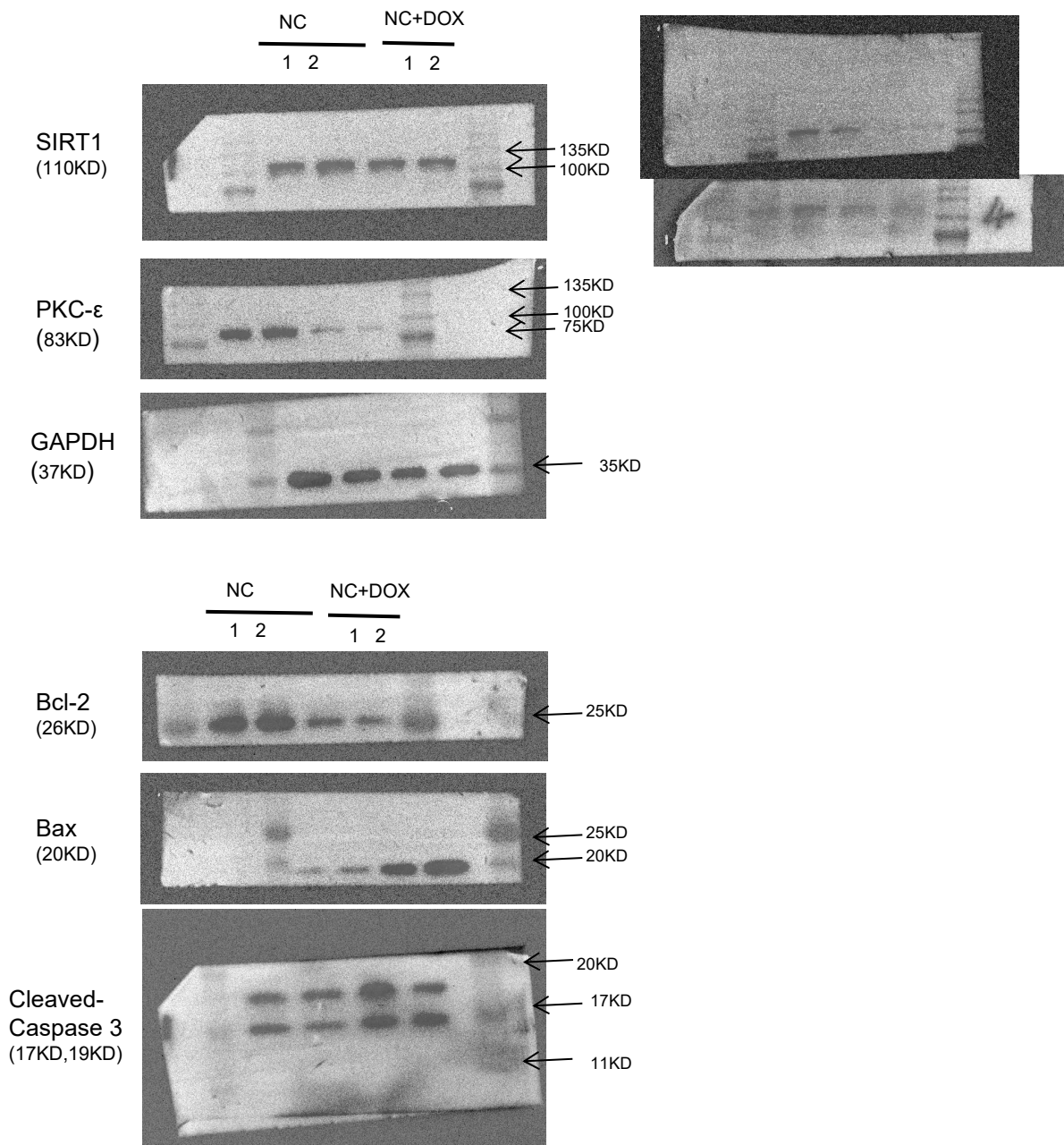

**Fig4**

IP

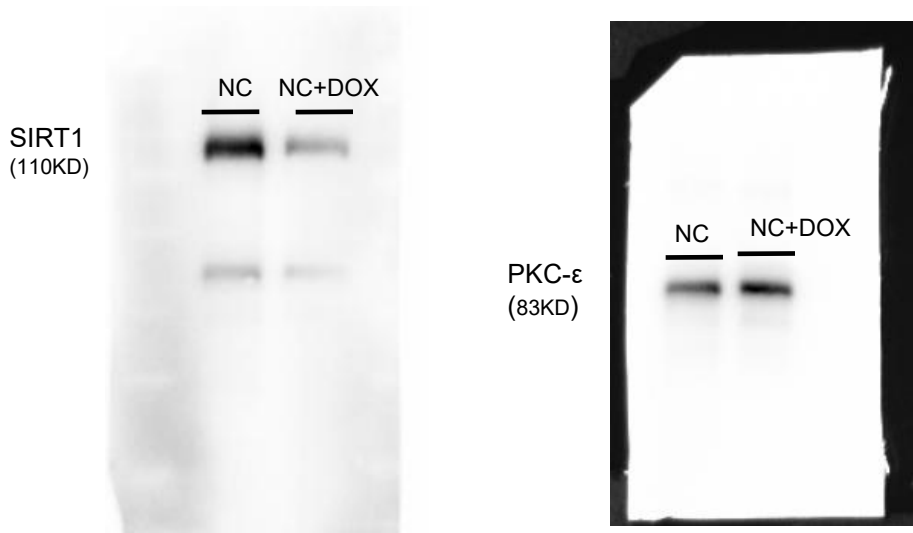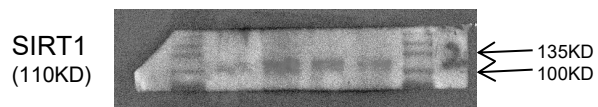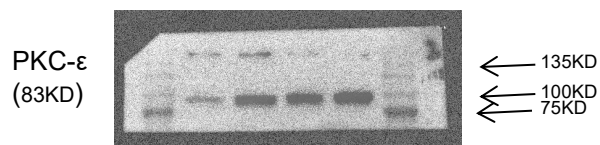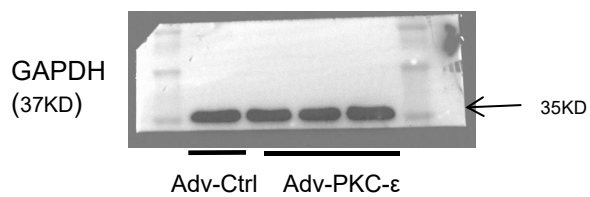

**Fig5**

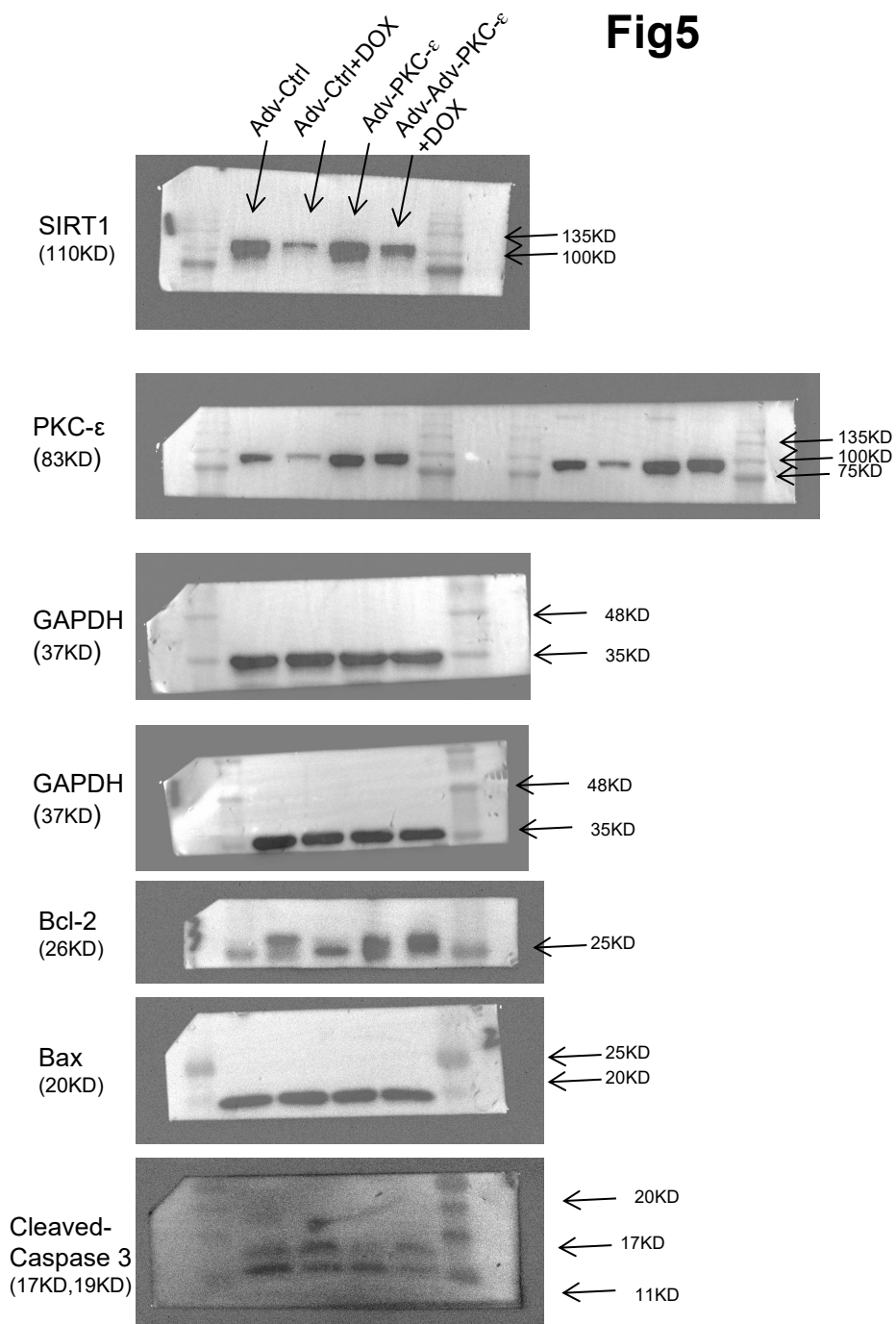

Fig6

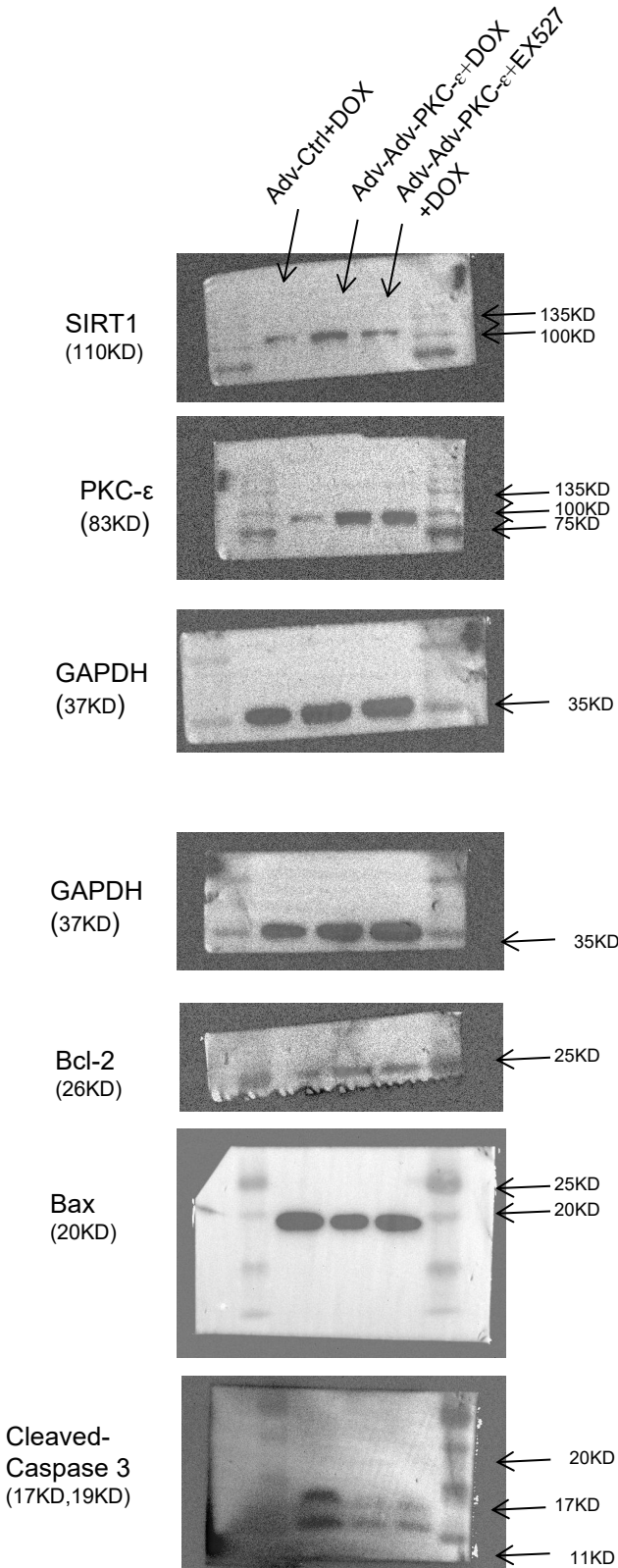

Supplement: Supplementary file 2 — Supplementary file2 (PDF 1510 KB) [file 12012_2025_9995_MOESM2_ESM.pdf]
